# A paired dataset of T1- and T2-weighted MRI at 3 Tesla and 7 Tesla

**DOI:** 10.1038/s41597-023-02400-y

**Published:** 2023-07-27

**Authors:** Xiaoyang Chen, Liangqiong Qu, Yifang Xie, Sahar Ahmad, Pew-Thian Yap

**Affiliations:** 1 Department of Radiology, University of North Carolina, Chapel Hill, NC 27599 USA; 2 Biomedical Research Imaging Center (BRIC), University of North Carolina, Chapel Hill, NC 27599 USA; 3 McAllister Heart Institute, University of North Carolina, Chapel Hill, NC 27599 USA

**Keywords:** Computational neuroscience, Research data

## Abstract

Brain magnetic resonance imaging (MRI) provides detailed soft tissue contrasts that are critical for disease diagnosis and neuroscience research. Higher MRI resolution typically comes at the cost of signal-to-noise ratio (SNR) and tissue contrast, particularly for more common 3 Tesla (3T) MRI scanners. At ultra-high magnetic field strength, 7 Tesla (7T) MRI allows for higher resolution with greater tissue contrast and SNR. However, the prohibitively high costs of 7T MRI scanners deter their widespread adoption in clinical and research centers. To obtain higher-quality images without 7T MRI scanners, algorithms that can synthesize 7T MR images from 3T MR images are under active development. Here, we make available a dataset of paired T1-weighted and T2-weighted MR images at 3T and 7T of 10 healthy subjects to facilitate the development and evaluation of 3T-to-7T MR image synthesis models. The quality of the dataset is assessed using image quality metrics implemented in MRIQC.

## Background & Summary

Magnetic resonance imaging (MRI) is a non-invasive imaging technique that provides excellent soft tissue contrast. The three-dimensional (3D) MRI scans of the human brain offer detailed insights into its structure, crucial for (i) quantifying brain morphology; (ii) studying brain development; and (iii) diagnosing neurodegenerative and neurodevelopmental disorders.

**Fig. 1:**
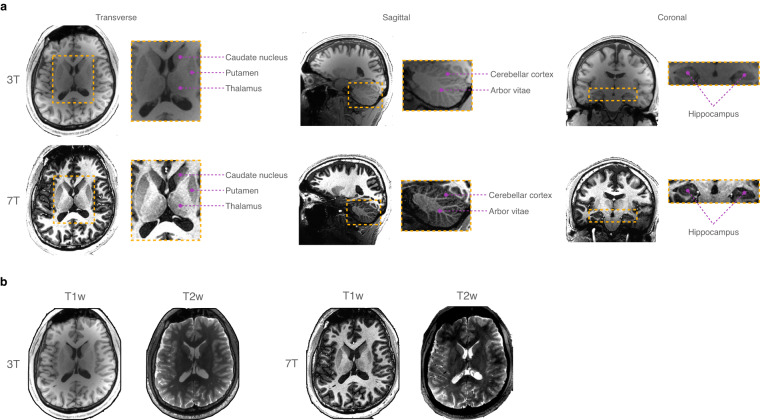
Paired T1w and T2w MRI scans at 3T and 7T acquired for a subject, shown for (**a**) multiple views and (**b**) multiple modalities.

The quality of images acquired with an MRI scanner depends largely on the strength of its magnetic field. In clinical settings, MRI scans are typically acquired with field strengths ranging between 0.2T and 3T. However, with clearance from regulatory bodies, 7T MRI scanners are now finding more clinical utility. A 7T MRI scanner yields higher spatial resolution and signal-to-noise ratio (SNR) than a 3T MRI scanner, giving improved anatomical visibility with enhanced contrast between gray matter (GM) and white matter (WM) tissues (Fig. 1a). This allows more fine-grained characterization of cortical folds and subcortical regions, facilitating MRI postprocessing such as tissue segmentation, anatomical parcellation, and cortical/subcortical surface reconstruction. Moreover, 7T MRI has demonstrated clinical utility in revealing subtle abnormalities due to pathological conditions^1–3^ that are elusive in low-field MRI scans. Despite advantageous to research and clinical studies, 7T MRI scanners are not widely adopted due to its high cost, complicated maintenance, and skill requirements in image acquisition and interpretation^4^. There are only about 100 7T MRI scanners, compared to 20,000 3T scanners, worldwide^5^. Additionally, such advanced imaging technology is typically inaccessible to low- and middle-income countries.

For low-cost MRI quality improvement, 7T images can be synthesized from 3T images to enhance tissue contrasts and anatomical details for improved downstream processing and analysis. In recent years, many deep learning based 3T-to-7T image synthesis methods have been proposed^5–7^. Typically, a convolutional neural network (CNN) is used to learn a non-linear 3T-to-7T mapping using paired 3T and 7T images acquired from the same subjects. Deep learning using adversarial training^6^, cascaded regression^5^, and image priors^7^ has been demonstrated to generate realistic 7T images from their 3T counterparts. However, obtaining paired images for training can be challenging in practice. While CycleGAN-based image synthesis models^8^ can still be trained in the absence of paired images, paired data is still critical for quantitative model evaluation.

Here, we disseminate a dataset of paired T1-weighted (T1w) and T2-weighted (T2w) brain MRI scans acquired at 3T and 7T. We provide a comprehensive description of the design, acquisition, and preparation of the dataset. Image quality is assessed using quality metrics implemented in MRIQC^9^. We expect that this dataset will serve as a valuable resource for research and development in 3T-to-7T and T1-to-T2 image synthesis.

## Methods

### Participants

The MRI data was collected for 10 healthy adult volunteers (3 females and 7 males; age range: 25–41 years; median age: 32.0 years; IQR: 11.0 years) with no reported history of neurological or psychiatric diseases. All participants provided written informed consent before participation. The study protocols were approved by the Institutional Review Board of the School of Medicine of the University of North Carolina at Chapel Hill.

### Image acquisition

The brain MRI scans were acquired using 3T Siemens Magnetom Prisma and 7T Siemens Magnetom Terra scanners, equipped with 32-channel head coils, using the following sequences (i) 3T T1w MPRAGE with 208 sagittal slices, repetition time (TR) = 2,400 ms, echo time (TE) = 2.2 ms, flip angle (FA) = 8°, acquisition matrix = 320 × 320, and resolution = 0.8 × 0.8 × 0.8 mm^3^. (ii) 3T T2w SPACE with 208 sagittal slices, TR = 3,200 ms, TE = 563 ms, acquisition matrix = 320 × 320, and resolution = 0.8 × 0.8 × 0.8 mm^3^. (iii) 7T T1w MP2RAGE with 256 sagittal slices, TR = 6,000 ms, TE = 1.91 ms, FA_1_ = 4°, FA_2_ = 4°, acquisition matrix = 304 × 308, and resolution = 0.65 × 0.65 × 0.65 mm^3^. (iv) 7T T2w turbo spin echo with 252 sagittal slices, TR = 3,000 ms, TE = 282 ms, FA_1_ = 120°, FA_2_ = 7°, acquisition matrix = 320 × 208, and resolution = 0.65 × 0.65 × 0.65 mm^3^.

### Image processing

**Fig. 2:**
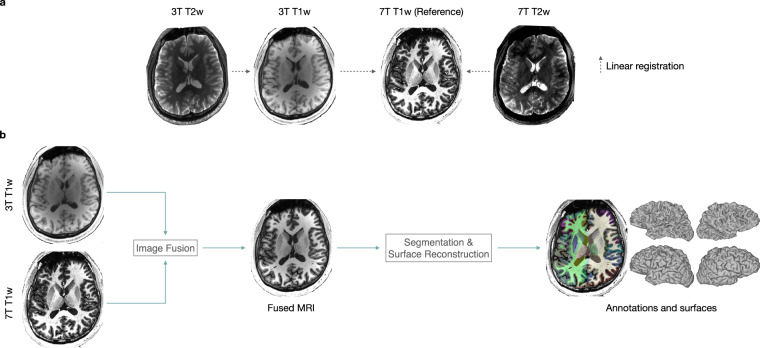
Data processing: (**a**) Linear registration of 3T T1w, 3T T2w, and 7T T2w images to 7T T1w image. (**b**) Brain annotation and surface reconstruction.

**Fig. 3:**
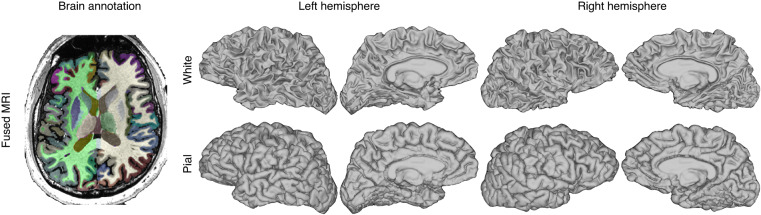
Cortical and subcortical annotations and white and pial cortical surfaces.

The acquired MRI scans were converted from DICOM to Neuroinformatics Informatics Technology Initiative (NIfTI) format using MRIcroGL (https://www.nitrc.org/projects/mricrogl) and then organized in accordance with the Brain Imaging Data Structure (BIDS)^10^ format. Facial information was removed from all the MRI scans using PyDeface^11^. Aligned images obtained with FSL FLIRT^12,13^ were also provided. For each subject, the 3T T1w and 7T T2w images were linearly registered to the 7T T1w image. The 3T T2w image was then aligned with the registered 3T T1w image (Fig. 2a). For consistent cortical/subcortical annotation and cortical surface reconstruction, FreeSurfer^14^ was employed on harmonic means of 7T T1w images and 3T T1w images (Fig. 2b). Example results are shown in Fig. 3.

## Data Records

The dataset was made available via a Figshare repository^15^. The 3D structural images were anonymized and organized according to the BIDS^10^ standard. For each subject, original (“Nifti”) and aligned (“Aligned”) images for both 3T (“ses-1”) and 7T (“ses-2”) were provided along with annotation maps as well as pial and white matter cortical surfaces. The volumetric and surface data were stored as NIfTI and GIFTI files, respectively. Age and sex information was included for each participant in the data file (participants.tsv) as per the BIDS standard. The image quality reports generated with MRIQC^9^ tool can be found in the BIDS directory, where for each scan, the MRIQC HTML report was name-matched with the scan name. For each scan, values of the image quality metrics (IQMs) were included in a JSON file.

## Technical Validation

**Fig. 4:**
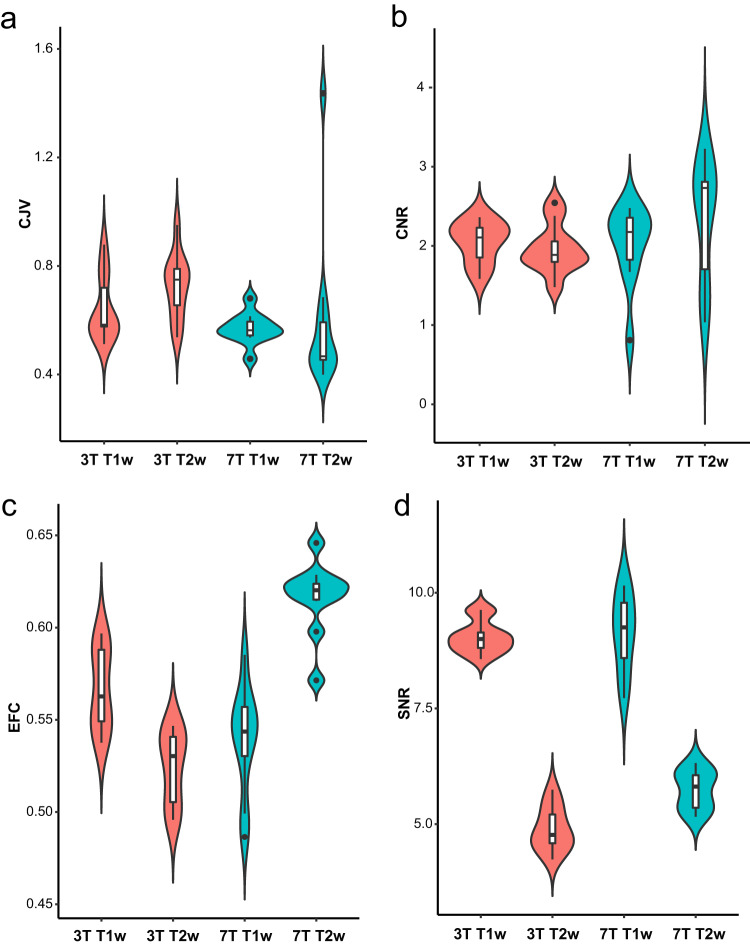
Violin plots for image quality metrics (IQMs) for different acquisition protocols: (**a**) coefficient of joint variation (CJV), (**b**) contrast to noise ratio (CNR), (**c**) entropy focus criterion (EFC), and (**d**) total signal-to-noise ratio (SNR).

We assessed image quality using four MRIQC IQMs (Fig. 4): (i) coefficient of joint variation (CJV; lower is better); (ii) contrast to noise ratio (CNR; higher is better); (iii) entropy focus criterion (EFC; lower is better); and (iv) total signal-to-noise ratio (SNR; higher is better). All images exhibit good tissue contrasts and structural details.

## Usage Notes

The dataset is available via Figshare. We encourage neuroscience researchers to use this dataset for their studies under the requirement of citing both the paper and the data. The unique feature of our dataset is that each participant has paired T1w and T2w MRI brain scans acquired at both 3T and 7T, cortical and subcortical annotations, and white and pial cortical surfaces.

## Data Availability

We processed the brain MRI data using open-source software packages: MRIcroGL (v20), PyDeface (v2.0.2)^11^, MRIQC (v22.0.6)^9^, FSL FLIRT (v6.0)^12,13^, and FreeSurfer v7.1.0^14^. No custom code was utilized.

## References

[1] Kerchner G (2010). Hippocampal CA1 apical neuropil atrophy in mild Alzheimer disease visualized with 7T MRI. Neurology.

[2] Cho Z-H (2010). Direct visualization of deep brain stimulation targets in parkinson disease with the use of 7-tesla magnetic resonance imaging. Journal of Neurosurgery.

[3] Radbruch A (2014). Quantification of tumor vessels in glioblastoma patients using time-of-flight angiography at 7 Tesla: a feasibility study. PloS one.

[4] Cosottini, M. & Roccatagliata, L. Neuroimaging at 7T: are we ready for clinical transition? (2021). 10.1186/s41747-021-00234-0 PMC8387509 34435257

[5] Zhang Y, Yap P-T, Qu L, Cheng J-Z, Shen D (2019). Dual-domain convolutional neural networks for improving structural information in 3T MRI. Magnetic Resonance Imaging.

[6] Nie D (2018). Medical image synthesis with deep convolutional adversarial networks. IEEE Transactions on Biomedical Engineering.

[7] Qu L, Zhang Y, Wang S, Yap P-T, Shen D (2020). Synthesized 7T MRI from 3T MRI via deep learning in spatial and wavelet domains. Medical Image Analysis.

[8] Do H, Bourdon P, Helbert D, Naudin M, Guillevin R (2021). 7T MRI super-resolution with generative adversarial network. Electronic Imaging.

[9] Esteban O (2017). MRIQC: Advancing the automatic prediction of image quality in MRI from unseen sites. PLOS ONE.

[10] Gorgolewski KJ (2016). The brain imaging data structure, a format for organizing and describing outputs of neuroimaging experiments. Sci. Data.

[11] Gulban OF (2022). Zenodo.

[12] Jenkinson M, Bannister P, Brady M, Smith S (2002). Improved optimization for the robust and accurate linear registration and motion correction of brain images. NeuroImage.

[13] Jenkinson M, Beckmann CF, Behrens TE, Woolrich MW, Smith SM (2012). FSL. NeuroImage.

[14] Fischl B (2012). FreeSurfer. NeuroImage.

[15] Chen X, Qu L, Xie Y, Ahmad S, Yap P-T (2023). Figshare.

